# Eight Weeks of Daily Cannabidiol Supplementation Improves Sleep Quality and Immune Cell Cytotoxicity

**DOI:** 10.3390/nu15194173

**Published:** 2023-09-27

**Authors:** Jacob N. Kisiolek, Victoria A. Flores, Arjun Ramani, Blake Butler, James M. Haughian, Laura K. Stewart

**Affiliations:** 1 Department of Kinesiology, Nutrition, and Dietetics, University of Northern Colorado, Greeley, CO 80639, USA; vflores1@uic.edu (V.A.F.); arjun.ramani@unco.edu (A.R.); butler.blake14@gmail.com (B.B.); 2 Department of Pathology, Division of Microbiology and Immunology, University of Utah, Salt Lake City, UT 84112, USA; 3 Department of Kinesiology and Nutrition, University of Illinois at Chicago, Chicago, IL 60612, USA; 4 Department of Biological Sciences, College of Natural and Health Sciences, University of Northern Colorado, Greeley, CO 80639, USA; james.haughian@unco.edu

**Keywords:** cannabidiol, natural killer cell, immunophenotype, sleep, sleep quality, mental health

## Abstract

Background: The endocannabinoid system is active in nervous and immune cells and involves the expression of two cannabinoid receptor genes (CB1 and CB2), along with endogenous endocannabinoid ligands, 2-arachidonoyl glycerol (2-AG) and arachidonoyl ethanolamide (anandamide), and their synthetic enzymes. Cannabidiol (CBD) is a non-intoxicating exogenous cannabinoid agonist derived from plants that, at high doses, has received FDA approval as an anticonvulsant for epileptic seizures, and at low doses is marketed as a food-grade supplement for improved mental health, sleep quality, and immunological function. At present, the predominance of published CBD clinical research has focused on ameliorative or disease-specific intervention, with few trials investigating CBD effects in healthy populations. Methods: This clinical study aimed to investigate the effects of 8 weeks of 50 mg oral CBD on mental health, sleep quantity and quality, and immune cell function in healthy, college-aged individuals. Twenty-eight participants (average age 25.9 ± 6.1 y) were randomized to receive either daily oral capsules of 50 mg of CBD (CB, *n* = 14) or a calorie-matched placebo (CN, *n* = 14). Participants completed pre- and post-intervention assessments, including anthropometric measurements, mental health surveys, sleep analysis, and immunological function assessments. Results: After completing the 8-week intervention, there were no significant changes in body weight and BMI (CN: 1.09 ± 0.89%: CB: 1.41 ± 1.07%), or body fat percentage (CN: 9.01 ± 7.51%: CB: 8.57 ± 7.81%), respectively (values are % change pre to post, *p* > 0.05). There were also no significant differences between CB and CN groups with respect to mental health measures, sleep quantity, or circulating immunophenotype as a result of the intervention. However, the CB group experienced significant improvements in sleep quality measured objectively using a sleep questionnaire (*p* = 0.0023) and enhanced Natural Killer (NK) immune cell function assessed in situ (*p* = 0.0125). Conclusions: Eight weeks of daily 50 mg CBD may improve sleep quality, and NK immunosurveillance in healthy, younger adults.

## 1. Introduction

Mental health challenges in the United States are on the rise. Since 2019, the proportion of men and women between the ages of 18 and 44 being treated for mental illness increased by 4.7 and 4.8%, respectively, [1]. In congruence with the rise in mental health challenges, sleep quality in the United States has declined, with one in three American adults reporting sleeping less than the 7 h per night minimum recommended to promote optimal health and general wellbeing [2]. This rise in mental illness and decline in sleep quantity, combined with the limited reach of the American health care system, creates a need for research into cost-effective and non-invasive strategies that may improve mental well-being and sleep quality in the general population, including the often-neglected younger adult population.

*Cannabis sativa* is a species of cannabis plant containing more than 140 cannabinoids that has been used medicinally since 2700 BC [3]. The *Cannabis sativa* plant synthesizes and concentrates phytocannabinoid molecules for the purpose of defense against predation [4]. The phytocannabinoids receiving significant attention because of their bioactivity within the human body include Δ9-tetrahydrocannabinol (Δ9-THC) and Cannabidiol (CBD). Δ9-THC drives recreational cannabis use as it is the primary intoxicating compound, where consumption results in feelings of euphoria or, more colloquially, the “high”. Conversely, CBD is a non-intoxicating molecule, and its consumption is anecdotally associated with improvements in mental health, sleep quality, and immunological function [4,5]. 

When present in the human blood circulation, the Cannabis-derived phytocannabinoids gain access to the endogenous cannabinoid or ‘endocannabinoid’ physiological system active throughout the body. The endocannabinoid system involves two cannabinoid receptors (CB1 and CB2), along with endogenously secreted ligands and their synthetic enzymes. These proteins are actively expressed in the nervous, immune, and other bodily systems, and are believed to be the primary target through which CBD and other exogenous cannabinoids exert their effects [4]. CBD is thought to function as a cannabinoid receptor agonist that is primarily active on CB2 receptors [6]. However, CBD has a complex pharmacology that includes direct or indirect actions on many other potential cellular targets: 5-HT1a, TRPA1, TRPV1, TRPV2, TRPV3, A2, and A1, with agonistic or inverse agonist effects reported [7]. Since the discovery of the endocannabinoid system in the 1990s, additional endogenous and exogenous ligands and receptors have been identified, all of which may have varying roles in mediating the effects of CBD within the human body.

CBD is touted as a mental health and sleep aid; however, as an exempt nutritional supplement, these claims have not been interrogated by the FDA, and clinical trials supporting these marketing claims are lacking. This is despite increased consumer interest stemming from the state-level legalization of cannabis and its derivatives for recreational and health-related use. The legitimacy of CBD’s potential as a biologically active, therapeutic molecule is underscored by the successful clinical trials that led to the FDA-approval of CBD as an anticonvulsant prescription drug marketed under the trade name Epidiolex^®^. When examining cannabis use more broadly, evidence suggests that individuals use cannabis for the relief of mental health symptoms, effects that are often attributed to CBD [7]. In both animal and human investigations, CBD exhibits anxiolytic properties that work through alterations in the limbic and paralimbic brain regions [8,9]. Furthermore, CBD plays an agonistic role on the serotonin 1a receptor (5-HT1a), a receptor associated with mood changes, anxiety, depression, and immune regulation [8,10,11,12]. However, observational clinical trials tracking cannabis use and mental health measures have reported routine use as a cause of adverse mental health conditions such as anxiety, psychosis, and depression [13,14]. In regard to CBD and sleep, a single study with healthy individuals reported that subjects consuming a single oral-gelatin capsule containing 160 mg CBD dose reported significantly greater sleep duration accompanied by decreased dream recall when compared with groups consuming a placebo (glucose), 10, 40, or 80 mg of CBD [15]. Additional clinical investigations relating CBD to improved sleep quality include two case studies: one in a pediatric patient with post-traumatic stress disorder consuming 25 mg CBD oil daily [16], and the other in four Parkinson’s patients consuming 75–300 mg/day of CBD for 6 weeks [17]. 

The immune system plays a central role in regulating both mental health and sleep [18,19]. CBD is marketed as an anti-inflammatory compound, which may have valuable applications in both healthy and diseased populations [20]. CBD administration in a murine model is associated with significant reductions in pro-inflammatory cytokines IL-6 and TNF-α, an effect mediated through the agonism of the CB2 receptor [21]. Immune cells express CB2 at varying levels, with natural killer (NK) cells exhibiting some of the highest CB2 cell surface expression relative to other immune cells [22]. The number of circulating NK cells was significantly increased in adult male Wistar rats following 14 days of CBD administration (2.5 mg/kg or 5 mg/kg) [23], and CBD exposure in vitro was linked to improvements in Lymphokine-activated killer cells (LAK) cytotoxic activity [24]. However, these CBD-related effects on human immunity in NK cells have yet to be corroborated. 

Overall, considering the limited scope of randomized clinical trial data on CBD use as it pertains to mental health, sleep quality, and immune function in healthy individuals, we aimed to further investigate these outcomes in healthy young adults. We hypothesized that an 8-week CBD intervention would result in improved mental health outcomes, including reductions in symptoms of anxiety and depression, as well as enhanced sleep quantity and quality in healthy college-aged individuals. Additionally, we anticipated that the CBD intervention would be associated with an increase in NK cell number and improved NK cell function, reflecting a potential positive impact on immune regulation.

## 2. Materials and Methods

### 2.1. Participants

The participants in this study were recruited from the University of Northern Colorado and nearby communities. A total sample size of 28 participants was included in the current investigation. To be included, participants had to meet the following self-reported inclusion criteria: 18–50 years old, abstained from cannabis (Δ9-THC and/or CBD) for six weeks, and have no history of chronic alcohol and/or drug use. Participants were excluded if they were diagnosed with cardiovascular, neurological, metabolic, or mood disorders, were pregnant and/or nursing, or were unable to adhere to an 8-week supplement intervention. Furthermore, participants could not be regular users of anti-inflammatory medications or medications metabolized by the liver, nor could they be using sleep aids or sleep medications. Participants were excluded if they received the COVID-19 vaccination or were diagnosed with COVID-19 within three weeks of the initial visit. All available samples were used for this study. Prior to their involvement, all participants provided informed consent in accordance with the Declaration of Helsinki, and the protocol was approved was approved by the Institutional Review Board at the University of Northern Colorado (Protocol Number 2005001624A002, approved on 19 June 2020. This clinical trial was registered with clinicaltrials.gov (clinicaltrials.gov ID: NCT04881539. CBD, Immune Function, and Neural Health).

### 2.2. Experimental Design

**Figure A1:**
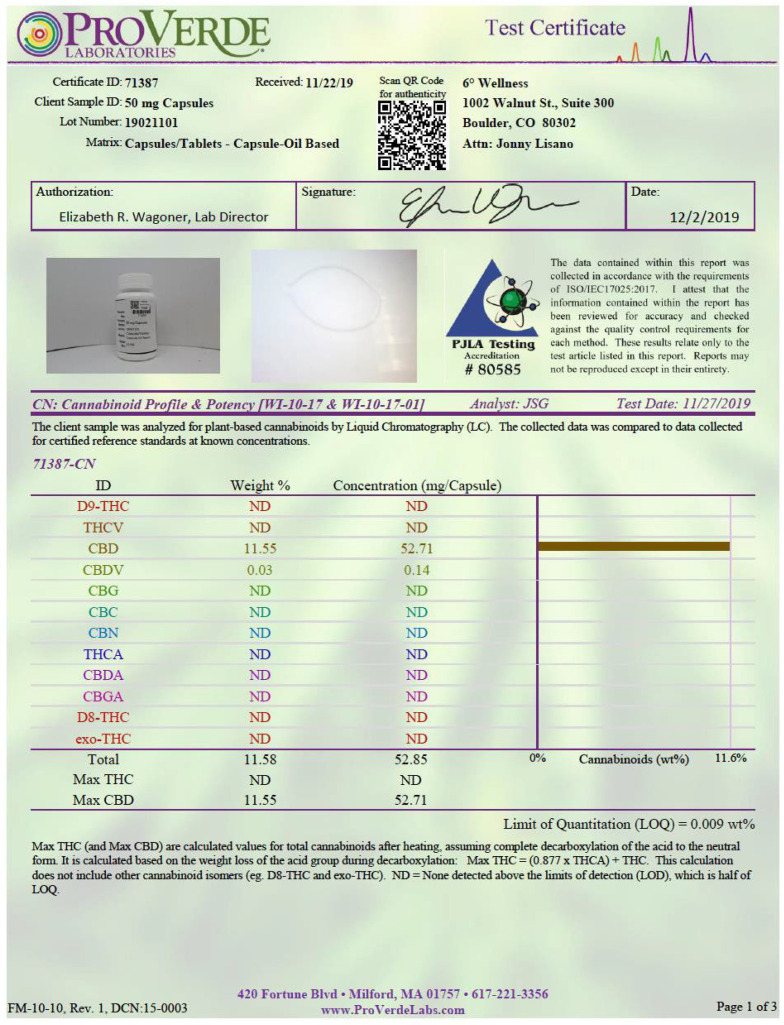

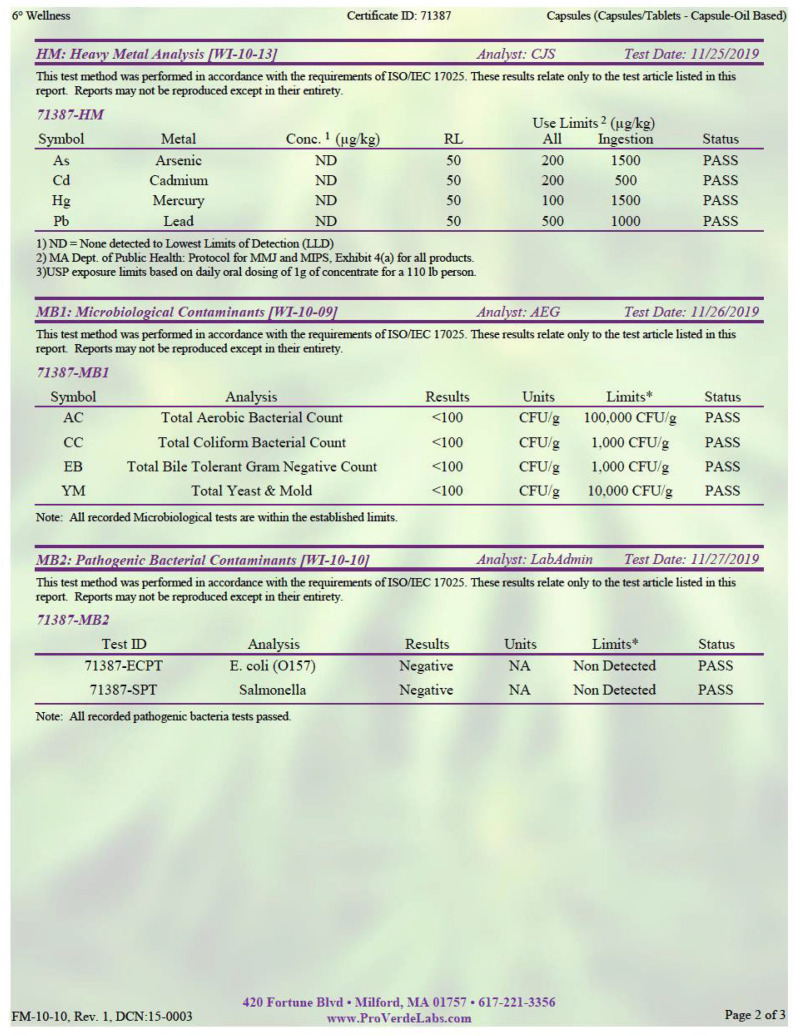

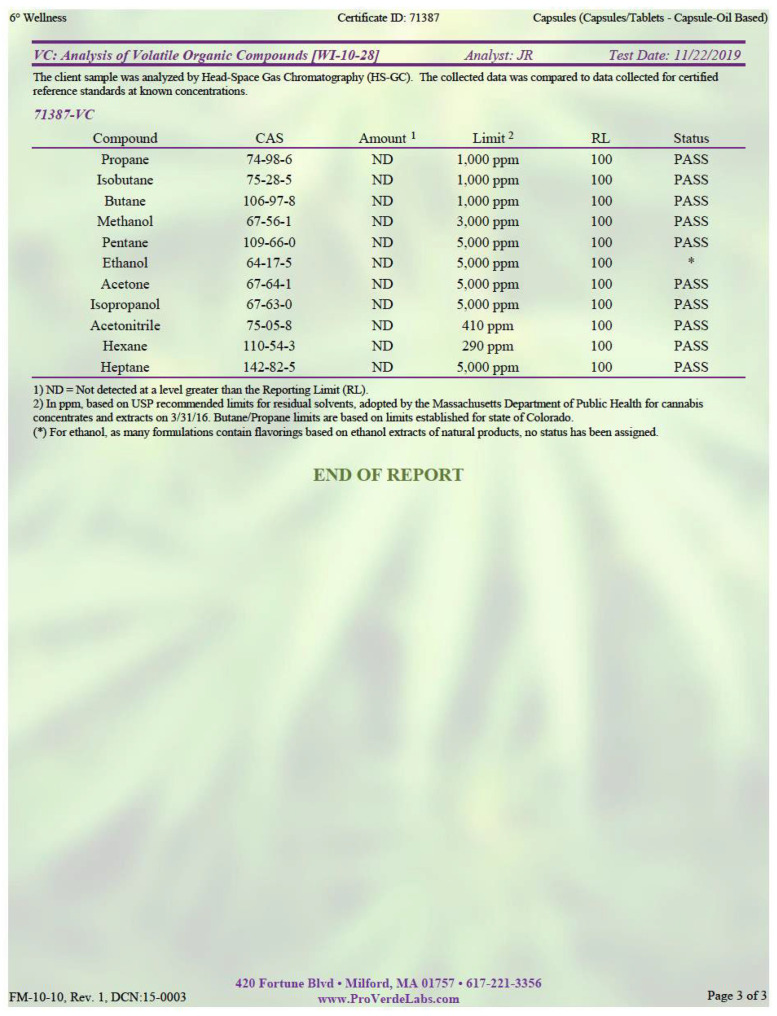
Third-Party Purity Analysis.

In this double-blind, placebo-controlled intervention, participants were randomized with the assistance of a third-party researcher not directly involved in any subject interaction, data collection, or sample processing, into one of two groups: the CBD group (CB: *n* = 14) or the calorie-matched placebo group (CN: *n* = 14). Subject randomization was accomplished through the excel randomization procedure and treatment assignment was never shared with data collection specialists as the trial was ongoing. Participants underwent two total visits separated by an 8-week intervention period. At the first visit, upon arrival, participants temperature was taken to ensure the absence of a fever, and a COVID-19 questionnaire was completed to ensure participants did not test positive for the COVID-19 virus or receive a vaccine 3 weeks prior to the start of the investigation and before the post intervention visits. Following the COVID-19 precautions, and after obtaining informed consent, participants completed a medical health history questionnaire and the Physical Activity Readiness Questionnaire (PARQ), to ensure they met all inclusion criteria. A trained phlebotomist then collected blood samples after an 8 h fast and 48 h rest period. Anthropometric measurements, body composition analysis, and questionnaires assessing mental health, sleep quantity, and sleep quality were also obtained. After the first visit, participants were provided with a wrist actigraphy sleep tracker (Fitbit Inspire Heart Rate Fitness Tracker, Fitbit, San Francisco, CA, USA) to wear for seven days before the 8-week intervention to assess sleep quantity and quality. They were instructed to wear the Fitbit for 7 days prior to the 8-week intervention and were reassessed for sleep quantity and quality during the second-to-last week (week 7) of the intervention period. Throughout the 8-week supplementation period, participants were instructed to consume orally one liquid gel pill per day containing either 50 mg of purified, hemp-derived CBD (Six Degrees Wellness, Boulder, CO, USA), or a 225 mg medium-chain triglyceride capsule (MCT; Nutiva, Point Richmond, CA, USA) as the calorie-matched placebo, following their last meal, 1–1.5 h before bed. Cannabidiol supplements were purchased from Six Degrees Wellness (Boulder, CO, USA) in liquid gel capsules that contained 50 mg of purified, hemp-derived CBD and 225 mg of coconut oil as a carrier (MCT; Nutiva, Point Richmond, CA, USA). Placebo supplements contained only 225 mg of coconut oil as a calorie-matched control. Liquid chromatography was performed by third-party analysis to ensure the purity and dosing of the CBD supplement (see Appendix A, Figure A1). A cross-sectional study conducted in 2021, investigating reasons for CBD use, determined that in a sample size of 387 participants, 54.4% of participants were taking a daily dose between 1 and 49 mg per day with capsules/pills being the second most common route of administration. Furthermore, of the 387 participants, 42.6% responded to using CBD for self-perceived anxiety, 37.5% used CBD for managing stress, and 37% used CBD for improving sleep [25]. Therefore, a dose of 50 mg per day of oral CBD was selected to match real world application of CBD consumption. In addition, most commercially available CBD products range from 15 to 50 mg of CBD per dose, and for these reasons, the selection of 50 mg CBD daily in the current investigation was intended to match commonly used doses and commercially available supplements. Furthermore, participants were instructed to consume one pill per day following their last meal, to optimize supplement bioavailability, as bioavailability of CBD decreases in a fasted state [26]. The third party, unaffiliated with data collection during the investigation, loaded all supplements into respective opaque pill boxes, and placed the boxes in opaque bags so that investigators remained blind to the intervention. Investigators met bi-weekly with participants to distribute two, 7-day pill boxes of their respective supplementation, as well as to ensure adherence to the supplementation protocol, and to obtain information related to any adverse side effects that occurred due to supplementation. At the end of the 8-week intervention, participants completed the post-intervention testing which consisted of the same measurements as the pre-intervention. It is important to note that this study was designed to explore a potential CBD-related mechanism of action. This manuscript represents a subset of data which was published more recently [27]. This subset of data was used due to our inability to collect immune-related outcomes for all randomized participants due to unforeseeable circumstances, and was in no way dependent or predicated on subject characteristics.

### 2.3. Anthropometric, Body Composition, and Physical Activity Assessment

Height and weight were obtained using a stadiometer SECA 220 (Chino, CA, USA) and the Detecto standing digital scale (Webb City, MI, USA), respectively. Body composition, lean body mass (LBM), and body fat percentage (BF%) were evaluated using air displacement plethysmography with a BODPOD (COSMED USA Inc., Concord, CA, USA). Participants were instructed to remove their shoes, socks, jewelry, and all additional clothing other than a base layer. This base layer was recorded, and participants were asked to return to the post-intervention measurement wearing the same attire. Participants were then given a swim cap to wear, and body composition analysis was performed via manufacturer’s guidelines [28]. Lastly, physical activity was assessed using the International Physical Activity Questionaire (IPAQ). The IPAQ is a physical activity questionnaire assessing amounts of differing levels of physical activity such as vigorous, moderate, walking, and sitting. Each section assess how many days per week in the past 7 days did the participant complete vigorous or moderate physical activity, in addition to how many days did they walk. Following questions on how many days per week, the IPAQ assesses how many minutes per day did they participate in the activity. Finally, the IPAQ assess how many hours did the participant spend sitting on an average week day. 

### 2.4. Measures of Mental Health

At pre- and post-intervention testing, participants were asked to complete the Beck’s Depression Inventory (BDI) [29], General Anxiety Disorder-7 (GAD-7) [30], Piper Fatigue Scale (PFS) [31], and Ferrans and Powers Quality of Life Index (QOL) [32] for assessment of their state of depression, anxiety, fatigue, and quality of life, respectively. Each questionnaire was given as a hard copy in the same order at pre- and post-intervention time points to ensure consistency between visits. 

The BDI is a 21-question depression assessment tool in which participants respond to questions with 0, 1, 2, or 3. Scores can range from 0 to 63, with higher scores associated with the presence of depression. The GAD-7 consists of seven questions assessing participants anxiety, with participants answering the seven questions on a 0–3 scale (0—“not at all”; 1—“several days”; 2—“more than half the days”; 3—“nearly every day”), with higher scores associated with the presence of anxiety. 

The PFS is a 27-question fatigue screening tool using a Likert based system (1–10) with “1” indicating little to no fatigue and “10” indicating maximal fatigue symptoms. Scores can range from 1 to 10. The PFS uses 22 of the 27 questions and is divided into four subscales that measure varying dimensions of fatigue including behavioral/severity, affective meaning, sensory, and cognitive/mood. The total scores and scores for each subscale were summed and totaled, then divided by the total number of responses to obtain fatigue scores. Lower total scores indicate reduced or minimal fatigue. 

The QOL is a 2-part questionnaire, totaling 66 questions. Part 1 asks how satisfied the participant is in various portions of their life including but not limited to; “Your health”, “Your family’s health”, and “The emotional support you get from your family?” Available responses range between 1 and 6 with “1” indicating “Very dissatisfied” and “6” indicating “Very satisfied.” Part 2 of the QOL asks the participant how important various portions of their life is to them. Questions in part 2 are identical to the questions in part 1, but the available responses range between 1 and 6 with “1” indicating “Very unimportant” and “6” indicating “Very important.” For assessment of the QOL index, 3.5 was subtracted from the raw scores of the first 33 questions assessing how satisfied they are with various aspects of their life. Next, these values were multiplied by their corresponding scores of the second 33 questions assessing how important various aspects of their life were to them. Finally, all 33 modified answers were summed up and divided by total number of responses followed by the addition of 15. This process created a range of values between 0 and 30, with higher values indicating higher QOL, and was suggested for optimal analysis by the creators of the survey [32]. Furthermore, the QOL can be separated into four subscales including health and functioning, social and economic, psychological/spiritual, and family. Analysis of the four subscales is completed exactly as stated above, just using questions that are specific for each subscale. Higher total scores indicate a better quality of life.

### 2.5. Leeds Sleep Evaluation Questionnaire

Participants were asked to report on their perceived sleep quality using the Leeds Sleep Evaluation Questionnaire (LSEQ) at pre- and post-intervention time points. The LSEQ is a 10-question visual analog scale (VAS) divided into four sections assessing “getting to sleep” (GTS), “quality of sleep” (QOS), “awake following sleep” (AFS), and “behavior following wakening” (BFW). Participants were instructed to mark a vertical tick mark along a 100 mm horizontal line closer or further away from their answer. The location of the tick mark was then measured and quantified as a quantitative millimeter value out of 100, with values closer to 100 associated with better quality of sleep.

### 2.6. Wrist Actigraphy Sleep Assessment

Participants were given a wrist actigraphy sleep tracker (Fitbit Inspire Heart Rate Fitness Tracker, Fitbit, San Francisco, CA, USA) to determine sleep quantity and quality at the initial visit, seven days prior to beginning the supplement intervention, and received a wrist actigraphy sleep tracker again at week seven, the final week of the intervention period. Participants were instructed to wear the wrist actigraphy sleep tracker at all hours of the day and night for one full week for pre- and post-intervention analysis. The face of the actigraphy band was covered with black electrical tape so that the participant could not receive immediate feedback about sleep performance.

### 2.7. Blood Sampling

Two separate blood samples at pre- and post-intervention time points were collected from all participants by a trained phlebotomist for the determination of immunophenotype and cytotoxic function. Participants were instructed to be at least 8 h fasted and all blood samples were collected between 0600 h and 1100 h. Post-intervention blood draws were conducted at the same time as the pre-intervention draw. Prior to the pre-intervention blood draw, participants were asked to complete a 24 h diet log which they were asked to replicate 24 h prior to the post-intervention blood draw. Participants were required to arrive rested, without strenuous physical exercise during the previous 48 h, and 24 h fasted from caffeine and alcohol. Participants donated approximately 10 mL of blood through venipuncture from the antecubital vein of the forearm at each time point. Blood was collected into vacuum sealed Becton, Dickinson and Company EDTA vials (Franklin Lakes, NJ, USA), and were immediately processed to assess immunophenotype and cytotoxic function.

### 2.8. Cell Culture

The K562 cell line (ATCC, Manassas, VA, USA), originally derived from a 53-year-old female patient with chronic myeloid leukemia in blast crisis [33], was used to evaluate cytotoxic function. One week prior to cytotoxic functional assays, a vial of K562 cells suspended in liquid nitrogen for long-term storage was thawed and handled under aseptic conditions. The thawed vial of K562 cells was transferred from the cryovial into a 15 mL conical tube (Corning, Tewksbury, MA, USA) containing 9.0 mL of pre-warmed complete cell culture medium (RPMI-1640; 10% FBS; 1% Pen-Strep). Cells were then spun down at 125× *g* for 10 min using a centrifuge (Eppendorf 5810R; Hamburg, Germany). After a pellet of K562 cells was formed, the cells were resuspended in complete cell culture medium at a concentration of 5.0 × 10^5^ cells/mL and added to a 75 cm^2^ culture flask with a vented cap (Fisher Scientific, Waltham, MA, USA). Cells were then incubated at 37 °C in a cell incubator at 5% CO_2_ for seven days, with replacement of media, and passaging of cells to 30–50% occurring every two days to ensure cell health and viability until use.

### 2.9. Peripheral Blood Mononuclear Cell Extraction

Fresh blood was used for the extraction of PBMC to ensure high viability of the cell sample. Ficoll–Paque density gradient media (15 mL) (GE Healthcare Bio-Sciences AB, Uppsala, Sweden) was added to a 50 mL conical tube. In a separate 15 mL conical tube, 7–10 mL of fresh whole blood was mixed with Dulbecco’s Phosphate Buffered Saline (DPBS) in a 1:1 ratio and inverted four times to mix. The blood:DPBS mixture was then slowly layered above the Ficoll–Paque density gradient media to prevent mixing. Once layered, the 50 mL conical tube containing fresh blood and Ficoll–Paque density gradient media was spun down at 400× *g* for 40 min with the centrifuge brake off. Once spun, the plasma layer of the solution was removed, and the buffy coat was extracted and placed in a conical tube. The removed buffy coat was then mixed with DPBS at 3× volume of the buffy coat (1 mL buffy coat: 3 mL DPBS) to wash cells. Cells were then spun down at 400× *g* for five minutes. The wash step was repeated three times. Then, cells were counted using a Countess 3 Automated Cell Counter (ThermoFisher Scientific; Waltham, MA, USA) and cells were then resuspended at a concentration of 1.0 × 10^6^ cells/mL. PBMC were removed and immediately used for phenotyping.

### 2.10. Immunophenotype and Cytotoxic Functional Analysis

Flasks of K562 cells were removed from the cell incubator and transferred into a conical tube. Cells were counted using a Countess 3 Automated Cell Counter (Invitrogen, Waltham, MA, USA) and spun down at 400× *g* for 10 min and resuspended in complete cell media at a concentration of 1.0 × 10^6^ cells/mL. A sample of 700 µL of K562 cells (7.0 × 105 total cells) were placed in a 2.0 mL Eppendorf tube (Eppendorf AG, Hamburg, Germany), and incubated with 2 µL of Alexa Fluor 647 conjugated anti-CD71 (Clone:CY1G4; Biolegend, San Diego, CA, USA) and 2 µL of Calcein-AM (eBioscience, Inc., San Diego, CA, USA) and placed on ice in the dark for 20 min. Following the incubation, K562 cells were washed, and spun down using a microcentrifuge (Eppendorf AG, Hamburg, Germany) at 400× *g* for 8 min and resuspended in complete cell culture media. Cells were then cultured alone (K562 only; control) at 1.0 × 10^5^ or co-cultured at an effector: target (E:T) cell ratio of 1:1, 5:1, 10:1, and 20:1. The five cell cultures were then incubated at 37 °C in a cell incubator at 5% CO_2_ atmosphere for 4 h. Following the 4 h incubation, cells were removed, and spun down at 400× *g* for 8 min. Cells were then resuspended in 0.2 mL of DPBS and incubated with 2 µL of eFluor 450 conjugated anti-CD3 (Clone:SK7; Invitrogen, Carlsbad, CA, USA) and 2 µL of PE-eFluor 610 conjugated anti-CD56 (Clone: CMSSB; Invitrogen, Carlsbad, CA, USA) on ice in the dark for 20 min. Following incubation, cells were again spun down as described above, resuspended in DPBS, and analyzed for immunophenotype and cytotoxic function using the Attune™ NxT Flow Cytometer (ThermoFisher Scientific, Carlsbad, CA, USA). 

Analysis of acquired raw data was performed using FlowJo software (version 10, TreeStar, Ashland, OR, USA). Cell cultures containing only PBMC populations were used for immunophenotyping. Cell populations were determined as being CD3^+^CD56^+^, CD3^+^CD56^−^, CD3^−^CD56^+^, and CD3^−^CD56^++^. Cytotoxic function was assessed using median fluorescence intensity of K562 human leukemia cells positive for the cell-permeant dye calcein-AM (eBioscience, Inc., San Diego, CA, USA). Calcein-AM is a nonfluorescent cell-permeant dye capable of freely passing into a cell’s cytosol. In live cells, calcein-AM is converted to a green fluorescent calcein by intracellular esterase performing acetoxymethyl ester hydrolysis [34]. If a cell stained with calcein-AM expresses a positive event, it is considered to be a live cell; however, once cells containing green fluorescent calcein undergo cellular death, calcein no longer exists in the cell and is considered a dead cell. K562 leukemia cells were cultured alone as a control for cell viability. 

Data were processed as normalized to the control K562-only culture for each blood sample. Median fluorescence intensities of 1:1, 5:1, 10:1, and 20:1 ratios were divided by the K562-only control median fluorescence intensity, then multiplied by 100 to normalize data to the control cell viability of K562 only and expressed as percent cell viability as previously shown [35].

### 2.11. Statistical Analyses

To avoid Type I error and achieve a desired level of 0.95 power with an α = 0.05, a priori analysis (G*Power, Dusseldorf, Germany) based on cytotoxicity changes observed by Specter et al. [36] in PBMC treated with THC showed that a total sample size of at least eight per group was needed. All data were evaluated for normality using the Shapiro–Wilk’s test (*p* > 0.05) and homogeneity of variance was assessed using Levene’s test for equality (0.483) prior to analysis. All data are presented as mean ± SD. To determine differences in the outcome means of each group at the pre-intervention time point, a Studentized *t*-test was conducted. Furthermore, a repeated measures ANOVA was conducted to examine the effects of CBD on all outcome measures over time to determine main effect outcomes including group, time, and a group-by-time interaction. All data expressed as a percentage were expressed as a proportion, and log transformed prior to statistical analysis. Significance was set at *p* < 0.05 and all statistical analyses were performed using SPSS 25 (IBM, Corp., Chicago, IL, USA) and GraphPad Prism 10 (GraphPad Software, San Diego, CA, USA).

## 3. Results

### 3.1. Study Participants

**Figure 1:**
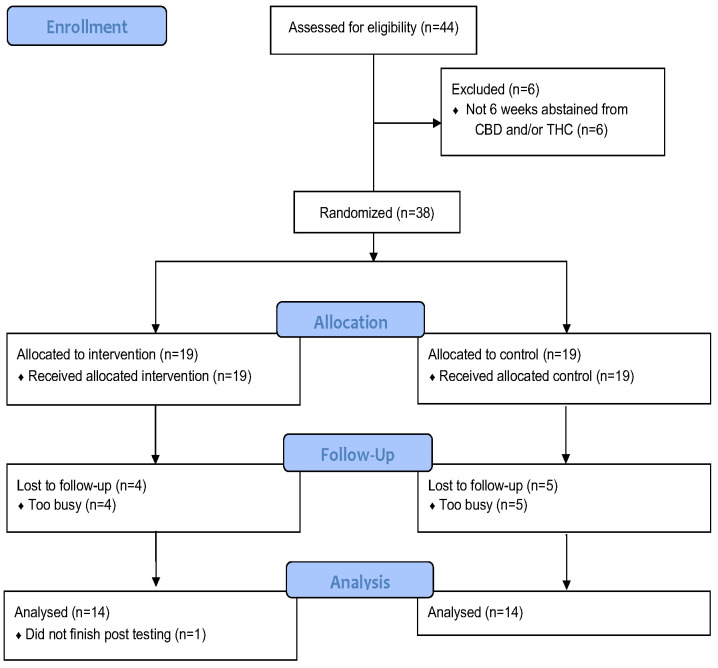
Consort Flow Diagram. CBD cannabidiol, THC tetrahydrocannabinol.

From (November 2020) to (February 2022), a total of twenty-eight individuals (CB, six males and eight females; CN eight males and six females) completed this study (Figure 1) with a combined average age of 25.9 ± 6.1 y. All participants completed the trial and were assessed for anthropometric, body composition, mental health, sleep, immunophenotype, and cellular cytotoxicity measures. The number of days in which participants performed vigorous physical activity were significantly different between CB and CN (*p =* 0.037) with the CB group participating in vigorous activity 33% more than CN. When baseline participant characteristics were seperated between biological sex regardless of intervention group, there were significant differences between males are females in height (*p =* 0.0002), weight (*p* = 0.005), LBM (*p* < 0.0001), and BF% (*p* < 0.0001). All the additional baseline characteristics of the males and females, and CB and CN groups at the pre-intervention time point, were the same for all other endpoints measured (Table 1), and there were no significant group or time main effects, or group × time interactions, consistent with successful pre-intervention randomization. At the pre-intervention time point, participants across all groups averaged BMIs of 24.7 ± 3.2 kg/m^2^ and a BF% of 20.4 ± 8.9%. Height, weight, and LBM were 170.4 ± 9.7 cm, 72.1 ± 12.5 kg, and 57.2 ± 11.3 kg, respectively.

### 3.2. Anthropometric Measures and Body Composition Assessment

Comparing CB and CN groups between the post-intervention time points, there were no significant group or time main effects, or group × time interactions, with regards to body composition. Table 2 represents post-intervention values. The percent change pre–post represents the change in each individual over time presented as mean ± SD. The mean percent change for CB and CN represents the change in mean values (CB pre mean to CB post mean; CN pre mean to CN post mean), with negative values representing a decrease in mean value pre- to post-intervention.

### 3.3. Measures of Mental Health

Table 3 represents mental health scores at post-intervention time points for CB and CN. Percent change pre–post represents the change in each individual over time presented as mean ± SD. The mean percent change for CB and CN represents the change in mean values (CB pre mean to CB post mean; CN pre mean to CN post mean), with negative values representing a decrease in mean value pre to post intervention. There were no significant group or time main effects or group × time interactions with respect to participant mental health as determined by BDI, GAD-7, PFS, or QOL scores.

### 3.4. Leeds Sleep Evaluation Questionnaire and Wrist Actigraphy

**Figure 2:**
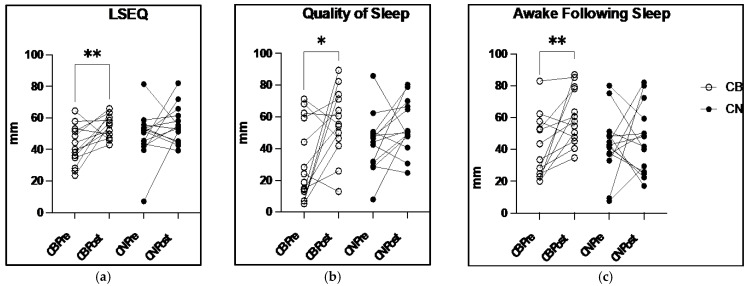
Leeds Sleep Evaluation Questionnaire (LSEQ) and subscales measured on a visual analog scale: (**a**) LSEQ mean score pre to post 8-week CBD intervention: (**b**) Quality of Sleep subscale of the LSEQ score pre to post 8-week CBD intervention; (**c**) Awake Following Sleep subscale of the LSEQ score pre to post 8-week CBD intervention; * indicates *p* < 0.05; ** indicates *p* < 0.01, mm millimeters.

There were no significant group or time main effects, or group × time interactions, in the LSEQ subscales of GTS or BFW. However, there was a significant main effect of time in the overall LSEQ scores (F_1,52_ = 9.6, *p* = 0.003) with a post hoc analysis demonstrating a significant 27.8% increase in LESQ between CB pre to CB post (Pre: 41.8 ± 12.1 mm Post: 54.9 ± 7.3 mm; *p* = 0.04), consistent with improved sleep quality. Additionally, a significant main effect of time was found for the LSEQ subscale AFS, which measures how awake participants feel following awakening (F_1,52_ = 5.469, *p* = 0.0232), with a post hoc analysis demonstrating a significant 53.9% increase between CB pre to CB post (Pre: 39.2 ± 19.7 mm Post: 60.4 ± 17.3 mm; *p* = 0.04). Furthermore, a significant group × time interaction was found in the LSEQ subscale QOS (F_1,52_ = 10.70, *p* = 0.002) with a post hoc analysis demonstrating a significant 79.2% increase between CB pre and CB post (Pre: 31.3 ± 24.6 mm Post: 56.0 ± 21.5 mm; *p* = 0.03). Despite changes in perceived sleep analysis (Figure 2), the analysis of wrist actigraphy sleep measurements showed no significant group or time main effects, or group × time interactions. When groups were combined, the overall mean ± SD were as follows: total minutes asleep (386.1 ± 59.8 min), minutes awake (52.1 ± 11 min), number of wake episodes (25.7 ± 7.3), time in bed (438.3 ± 68.1 min), and participant sleep efficiency (88.2 ± 1.7%). Table 4 presents LSEQ and wrist actigraphy analysis at post-intervention time points for CB and CN. The percent change pre–post represents the change in each individual over time presented as mean ± SD. The mean percent change for CB and CN represents the change in mean values (CB pre mean to CB post mean; CN pre mean to CN post mean), with negative values representing a decrease in mean value pre- to post-intervention.

### 3.5. Immunophenotype

**Figure 3:**
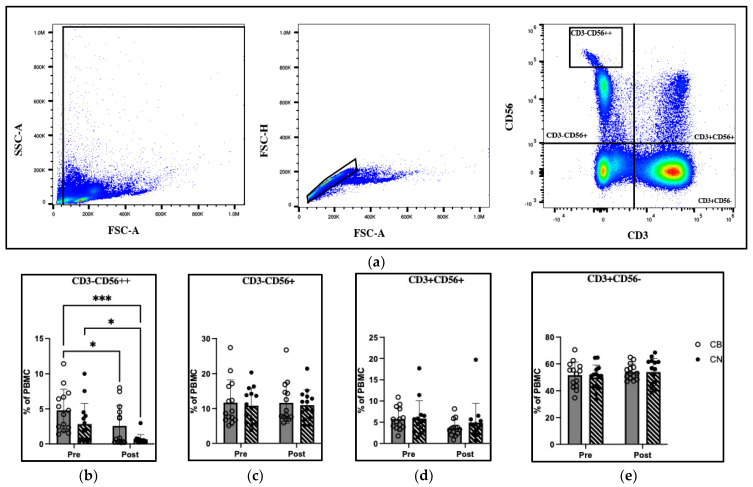
Immunophenotype analysis of peripheral blood mononuclear cells pre and post 8-week cannabidiol intervention (**a**) a visual representation of flow cytometry gaiting strategy; (**b**) CD3^−^ CD56^++^; (**c**) CD3^−^CD56^+^; (**d**) CD3^+^CD56^+^ (**e**) CD3^+^CD56^−^; % of PBMC percentage of total peripheral blood mononuclear cells, SSC-A Side Scatter Area, FSC-A Forward Scatter Area, FSC-H Forward Scatter Height. * Indicates *p* < 0.05; *** indicates *p* < 0.001.

The gating profile and changes in immune cell percentages in total PBMC are reported in Figure 3. There was a significant group (F_1,52_ = 8.74, *p* = 0.005) and time (F_1,52_ = 3.904, *p* = 0.0001) effect in the CD3^−^CD56^++^, CD56_bright_ NK cell population, with the post hoc analysis demonstrating a 46.5% decrease in cell percentage from CB pre to CB post (*p* = 0.01), and a 76.9% decrease in cell percentage from CN pre to CN Post (*p* = 0.03). Table 5 presents immunophenotype analysis at post-intervention time points for CB and CN. The percent change pre–post represents the change in each individual over time presented as mean ± SD. The mean percent change for CB and CN represents the change in mean values (CB pre mean to CB post mean; CN pre mean to CN post mean), with negative values representing a decrease in mean value pre- to post-intervention.

### 3.6. Cellular Cytotoxicity

**Figure 4:**
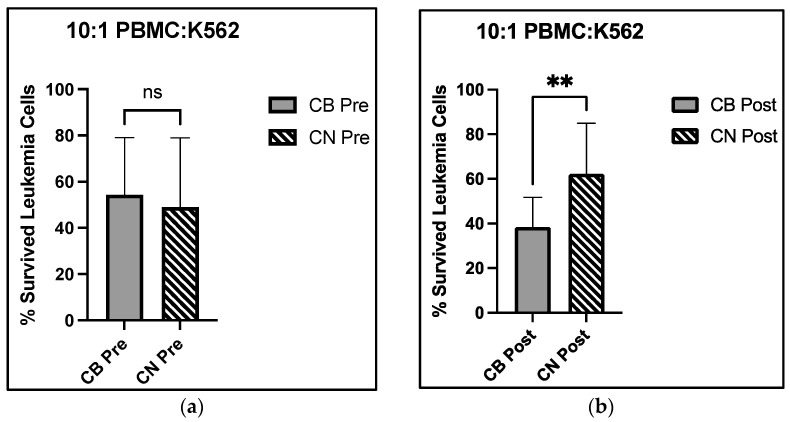

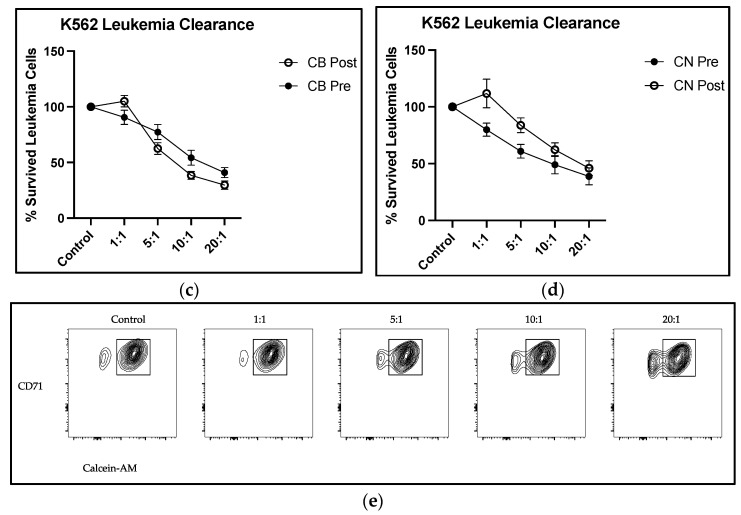
Cytotoxic analysis of PBMC towards K562 target cells pre and post 8-week cannabidiol intervention and (**a**) percent live K562 cell differences at the 10:1 E:T cell ratio at the pre-intervention time point; (**b**) percent live K562 cell differences at the 10:1 E:T cell ratio at the post-intervention time point; (**c**) pre to post percent live K562 cell changes in CB; (**d**) pre to post percent live K562 cell changes in CN; (**e**) flow cytometric representation of K562 cell viability decreasing as the E:T cell ratios increase; CB Cannabidiol, CN Control, Pre Pre intervention, Post Post intervention, % Survived Leukemia Cells percentage of live cancer cells; ** indicates *p* < 0.01; mm millimeters.

There was a significant main effect of time for MFI of 1:1 effector target cell ratio for cellular cytotoxicity (F_1,52_ = 8.278, *p* = 0.006) with post hoc analysis demonstrating a 39.8% reduction in surviving leukemia cells between CN pre and CN post (F_1,52_ = 8.278, *p* = 0.03). Furthermore, a significant group × time interaction was observed for MFI of 10:1 effector target cell ratio for cellular cytotoxicity (F_1,52_ = 6.689, *p* = 0.01) with post hoc analysis demonstrating a 23.8% reduction in surviving leukemia cells in CB post compared with CN post (*p* = 0.02), indicating a reduction in viable leukemia cells and enhanced NK cell function. There were no further group or time main effects, or group × time interactions for cellular cytotoxicity. Cytotoxicity functional analysis and flow outputs are reported in Figure 4.

## 4. Discussion

The present study was the first to investigate potential changes in mental health, perceived sleep, actigraphy-monitored sleep, immune cell distribution, and cytotoxic function following eight weeks of daily, low dose CBD ingestion in healthy college-aged individuals. We demonstrate that the daily ingestion of 50 mg CBD, 1–1.5 h before sleep onset, leads to significantly improved perceived sleep quality compared with a placebo control (Figure 2). Additionally, using flow cytometry and a cell-permeating fluorescent dye, we provide evidence that daily CBD supplementation for eight weeks enhances NK cell cytotoxicity against a malignant human leukemic cancer cell line. Collectively, these data suggest that eight weeks of CBD supplementation enhances perceived sleep and improves systemic immunosurveillance through the enhancement of NK cell cytotoxic function.

In this investigation, daily CBD ingestion over the course of eight weeks improves participants’ subjective feelings of their overall quality of sleep as evidenced by a 27.8% increase in CB LSEQ scores from pre- to post-intervention compared with a 14.3% increase in placebo-treated CN (Figure 2a). When specific aspects of sleep were evaluated, such as the subjective QOS score where participants rate the quality of their sleep upon waking, CB experienced a 79.2% increase in this score compared with a 24.5% increase in CN (Figure 2b), a 54% higher increase in this subjective sleep measure. Similarly, the measure of feeling rested after a bout of sleep, or AFS, had a 53.9% increase in AFS scores in CB compared with only a 7.4% increase in CN (Figure 2c). Despite the significant gains in the perceived quality of sleep, the wrist actigraphy sleep data were unchanged (Table 3). This pattern of perceived sleep quality improvements without improvements in sleep duration is consistent with previous CBD interventions [37]. Two weeks of daily supplementation of 2.5 mg of sublingual spray CBD in patients over the age of 18 with chronic pain significantly improved participant sleep quality without altering sleep duration [36]. More specifically, 36.9% of nights slept were determined as “good” quality sleep compared with 17.0% in the placebo (*p* < 0.05), with no changes found in duration of sleep (CBD: 6.4 ± 1.4 h; Placebo: 6.3 ± 1.6 h) [37]. One proposed mechanism for CBD-induced sedative effects, and in turn better sleep quality, is the central downregulation of the corticotropin-releasing hormone (CRH) gene and a resultant decrease in systemic cortisol, mediated directly through CB_1_ receptor expression on CRH neurons in the paraventricular nucleus [38,39,40]. The downregulation of the hypothalamic–pituitary–adrenal (HPA) axis increases sleep time and decreases awakening throughout the night, which may be a potential molecular mechanism for the improvements in sleep quality in this investigation. Furthermore, improvements in perceived sleep quality, without changes in sleep duration, are still considered to be beneficial for overall health. An investigation involving 1318 military recruits observed that having “good” perceived sleep quality during a period of sleep restriction resulted in no greater risk of respiratory infection when compared with groups with no sleep restriction and “good” perceived sleep quality [41]. However, sleep-restricted participants with poor sleep quality had a significantly greater risk of respiratory infection (*p* < 0.05), [41]. The significant correlation observed between perceived “good” sleep quality and a reduced risk of respiratory infection provides support for improved perceived sleep quality remaining beneficial to human health without changes in sleep duration.

Determining the precise physiological mechanisms underlying these perceived improvements in sleep, for example whether mediated centrally through the nervous system or peripherally through immune or other systems, was beyond the scope of this clinical study. In a companion study, which included some of these same participants, we found no effects on circulating markers of inflammation [27], suggesting that peripheral anti-inflammatory effects did not play a role. The improvements in sleep quality without changes in sleep duration may be attributed to alterations in sleep architecture. Cannabinoid 1 (CB_1_) receptors are present in various brain regions involved in the sleep–wake cycle [16,42]. Previous work in animal models demonstrates that the activation of the CB_1_ receptors increases slow wave and REM sleep while decreasing wakefulness [43]. Further investigations also indicate that antagonists to the CB_1_ receptor cause increased wakefulness with reductions in slow wave and REM sleep [44], further supporting the activation of CB_1_ receptors to improve sleep quality. Another possible explanation is that the wrist-located actigraphy performed here does not fully capture or appreciate the true quality of an individual’s sleep. The current gold standard diagnostic assay to objectively measure sleep quality is polysomnography [45], and our results suggest that CBD supplementation deserves investigation using these advanced measures. Therefore, it remains unclear as to the exact mechanism in which CBD interacts with the cannabinoid receptors to elicit improvements in perceived sleep quality in a healthy population, yet the outcomes remain beneficial for overall health.

There were no significant CBD-related changes in depression, anxiety, fatigue, or quality of life measures within either CB or CN groups. It is possible that this may be attributed to generally low levels of depression, anxiety, and fatigue along with high levels of quality of life reported by the study participants. The majority of the existing literature that associates CBD with improved mental health focuses on individuals with diagnosed mental health disorders [46,47]. In the present study, when groups were combined at the pre-intervention time point, the average scores for the BDI and GAD-7 were 4.5 ± 3.8 and 6.3 ± 6.0, which were categorized as “these ups and downs are considered normal” and “mild anxiety,” respectively. This indicates that participants experienced relatively low levels of depression and anxiety, which may have limited the potential for detectable improvements in our mental health outcomes. 

The COVID-19 pandemic occurred at the time of the current investigation. These unprecedented times resulted in extreme social, economic, and lifestyle modifications many participants had never experienced. These modifications, such as social isolation, economic distress, and disruptions to daily life, have been shown to alter the outcomes of this investigation such as sleep quality, sleep quantity, and measures of mental health. 

During the COVID-19 pandemic, 958 participants were surveyed, answering questions on their sleep patterns pre- and during lockdown. These participants were excluded if they were currently taking medications for sleep, or if they were diagnosed or believed to have had COVID-19, in line with the current investigation. Of these 958 participants surveyed, 16.1% of participants reported a decrease in sleep duration pre- to during lockdown, with 23.4% (*p* < 0.01) reporting that sleep quality worsened [48]. While not statistically significant, the CN group in the current investigation saw increases pre- to post-intervention in minutes awake (1.6%) and wake episodes (10%), representing lower quality sleep. Conversely, all other sleep variables for CN increased below a significant threshold. These results and the results of the current investigation indicate that our participant population may have been less affected by the COVID-19 pandemic than other populations. 

Similar to sleep quality and quantity, measures of mental health have been shown to significantly worsen during the COVID-19 pandemic. In a cross-sectional survey study, 2031 United States college students were assessed during the COVID-19 pandemic on measures of depression (Patient Health Questionnaire) and anxiety (GAD-7). The results indicate 48.14% of those surveyed showed moderate-to-severe levels of depression, and 38.48% showing moderate-to-severe levels of anxiety, with 71.26% reporting their stress/anxiety levels had increased during the pandemic [49]. The results of the current investigation, however, oppose these results through a lack of significant changes pre- to post-intervention. The lack of changes observed in perceived sleep, actigraphy-monitored sleep, and mental health in the CN group during the COVID-19 pandemic are puzzling based on the current body of literature; however, COVID-19 rules and regulations in the United States varied on a state to state basis adding potential variability between our participants and the current body of literature.

It is well documented that immune cells contain some of the highest numbers of CB_2_ receptor expression on their cell surface, with NK cells expressing the second highest number, superseded only by B-cells [22]. Endocannabinoid ligands to these receptors alter NK cell migration [50], and knockout of the CB_2_ receptor in mice was associated with increased NK cell migration into the tumor microenvironment and reduced tumor burden in non-small cell lung cancer models [51]. However, CBD-related alterations in human NK cell function from exogenous CBD exposure in situ remained unexplored until the present investigation. We demonstrated that CBD supplementation has no effect on the distribution of NK (CD3^−^CD56^+^), NKT (CD3^+^CD56^+^), or T-cell (CD3^+^CD56^−^) cell populations (Figure 3c–e). However, significant group and time effects were detected between CB pre- and CB post-, CN pre- and CN post-, and CB pre- and CN post-intervention in the CD56_bright_ NK cell (CD3^−^CD56^++^) population (Figure 3b). The activation of CB_2_ receptors by their endocannabinoid ligand shows increased NK cell migration in vitro [50]; however, specific NK cell subsets were not distinguished. Furthermore, the NK cell migration assay used was performed in vitro using Transwell™ inserts. This in vitro model is unable to distinguish in vivo if NK cells migrate into or out of the peripheral blood. Therefore, the 46.5% decrease and 76.9% decrease in CB and CN CD56_bright_ NK cells pre- to post-intervention may be an indication that specific subpopulations of NK cells have differing migratory capacity upon the binding of CB_2_ receptors.

Our data support enhanced immune cell cytotoxicity in the presence of CBD. Our assay mixed the leukemic K562 cell line with circulating PBMCs, a population where all immune cells are represented—NK, NKT, T, and B cells, for example. There was a significant group × time interaction at the 10:1 effector to target cell ratio, with a 29.2% decrease in live leukemic K562 cells from CB pre- to CB post-intervention, compared with a 27.1% increase in live leukemic K562 cells from CN pre- to post-intervention (Figure 4a,b). The group × time interaction revealed that at the 10:1 E:T cell ratio, CBD improved NK cell cytotoxicity towards leukemic K562 cells. Interestingly, we observed a main effect of time at the 1:1 E:T cell ratio, with a 15.9% increase in live leukemic K562 cell viability for the CB group and a 39.8% increase for the CN group. While we did not isolate subpopulations within the PBMCs, this assay is proposed to reflect NK cell cytotoxicity due to NKT cells (CD3^+^CD56^+^) requiring antigen presentation by non-classical MHC-CD1d and can only perform cytotoxic activity following activation [52]. Furthermore, upon activation, these cells have no cytotoxic activity towards leukemic K562 cells [52]. Furthermore, CD56_bright_ NK cells decreased pre- to post-intervention in both groups. Natural killer cells are commonly differentiated into CD56_bright_ and CD56_dim_, with CD56_bright_ most known for being prominent in tissues and regulators of immune response through the production of cytokines. CD56_dim_ NK cells are known to be more abundant in peripheral blood and regulators of cellular cytotoxicity [50]. Furthermore, CD56_dim_ cells have greater activity upon contact with target cells when compared with CD56_bright_ [53]. The CD56_bright_ populations experienced significant decreases in both groups pre- to post-intervention, and their cellular function in regard to cytotoxicity plays little to no role in the changes seen in cytotoxicity results. Furthermore, their lack of abundance in peripheral blood and function during this cell contact cytotoxic assay likely has no effect in the group × time interaction found in the viability of target leukemic K562 cells. The lack of significant change in the NK cell percentage of PBMCs suggests that CBD may be slightly improving NK cell cytotoxicity towards target leukemic K562 cells on an individual cell basis, with these effects becoming more prominent as the number of NK cells increase in cell culture. This improvement in NK cell cytotoxicity may derive from CBD actions on the CB_2_ receptor pathway. However, the current literature on the signal transduction mechanisms downstream of CBD binding the CB_2_ receptor is variable and may be cell-specific, with some demonstrating CBD working as a CB_2_ agonist, and others as an inverse agonist [6,54]. Inverse agonism is when a ligand binds to a receptor and stimulates a functional response opposite of the receptor agonist. Given that studies investigating tumoricidal activity in CB_2_ receptor knockout mice showed increased tumoricidal activity by NK cells and a decreased tumor burden when compared with wild type littermates [51], it is plausible that CBD may modify CB_2_ receptor activation such that NK cells experience a functional increase in their cellular cytotoxicity. However, the lack of group × time interaction at the 20:1 E:T cell ratio may be due to the large increase in effector cell number. The increase from 1.0 × 10^6^ to 2.0 × 10^6^ may have over saturated the target cells with effector cells negating these effects compared with the increase from 5.0 × 10^5^ to 1.0 × 10^6^, as shown in Figure 4c,d. Overall, the lack of change observed in the immune cell distribution of PBMCs and the significant decrease in live K562 cell group × time interaction at the 10:1 E:T cell ratios demonstrated that eight weeks of CBD supplementation may increase NK cell cytotoxic function.

In line with sleep quantity, quality, and mental health, COVID-19 can modulate factors of the immune system. The previous literature has demonstrated that CD56_dim_ NK cells significantly decrease in human peripherial blood in both mild and severe COVID-19 infection when compared with healthy controls (*p* < 0.0001) [55]. Furthermore, NK cell function assessed through cytotoxicity towards K562 leukemia cells was found to significantly decrease during COVID-19 infection (*p* < 0.05) with both NK cell number and function being fully restored 3 weeks post COVID-19 infection [55]. While COVID-19 infection can modulate NK cell distribution in periperial blood and their function, precautions taken in the current investigation to ensure participants were covid free was successfully demonstrated by the lack of significant changes in CD3^−^CD56^+^ cell populations in either group pre- to post-intervention and significant improvements in NK cell cytotoxicity.

It should be noted that the supplementation and placebo groups were not matched based on biological sex. As noted in Table 1, the CB group contained six males and eight females, where the CN group contained eight males and six females. To date, most studies investigating the effects of CBD administration on biological sex have been performed in rodent models, with four studies performed in humans. Three of the four studies investigating the effects of CBD between biological sex found no differences between subjective and physiological responses to recalling trauma [56] and plasma CBD concentrations [57,58]. However, one study administerd an acute dose of 25 mg CBD via MCT and found a significantly higher area under the curve over 24 h (*p* = 0.0499) in females compared with males, demonstrating enchanced bioavaiability [59]. These results demonstrate that females may be more seceptible to the effects of CBD when compared with males and should be noted for future investigation.

The improvements in cellular cytotoxicity post-intervention without changes in NK cell populations demonstrates that the daily consumption of 50 mg of CBD for eight weeks results in alterations in NK cell function, specifically in its cytotoxic capacity. However, it should be noted that this effect was only observed at a 10:1 E:T ratio, and not at the others tested. With the significant 29.2% improvement in CB at the 10:1 ratio, the addition of more cytotoxically able NK cells demonstrated greater improvements in cellular cytotoxic function through compounding effects. 

## 5. Limitations

The current investigation has several limitations that should be considered. Cannabidiol supplementation in humans has been found to be safe and tolerable with dosing as high as 25–50 mg/kg/day; comparably, this investigation dosage was, on average, 0.69 mg/kg/day [60]. This was the first investigation at the University of Northern Colorado to administer CBD; therefore, to prevent any adverse side effects caused by CBD consumption, lower dosing was selected. Therefore, additional investigations examining our outcomes following higher doses of CBD supplementation are needed. With subject safety being of the utmost importance, the recency of the national legalization of hemp production enables limited, stepwise research to be conducted on the safety and efficacy of higher doses of CBD, thus giving us pause when considering doses higher than 50 mg of CBD daily for eight weeks. Additionally, it is recognized that the biological availability of CBD can vary between sexes. However, due to the lack of CBD metabolite testing before and after the intervention, we are unable to fully understand the actual CBD availability in the participants. This limits our ability to draw definitive conclusions about CBD uptake and metabolism. Additionally, this investigation was conducted just after the height of the COVID-19 pandemic. While covid vaccination and/or diagnosis 3 weeks prior to the investigation was an exclusion criteria, covid can have long lasting effects on human physiology as a whole from the immune system to sleep architecture. Participants were met on a bi-weekly basis and, if they had covid, would have informed the investigation team. However, similar to other viruses, covid can be asymptomatic and participants may have been unaware of their illness. Therefore, the COVID-19 virus must be stated as a limitation to the current investigation. Furthermore, while there were no significant differences between groups in mental health variables, it is known that mental health had large variability between 2020 and 2022 during the COVID-19 pandemic. While the current investigation does not show significant changes in participants’ mental health, variability between subjects during this time should be noted as a limitation. Furthermore, participants in the current investigation were healthy college-aged individuals without preexisting health conditions or mental health disorders. Therefore, assessing the mental health of participants with few mental health disorders leaves little room for observable improvements in mental health outcomes pre- to post-intervention. The relatively low baseline levels of mental health symptoms may have masked potential changes in mental wellbeing. It will require further investigation into whether these results are applicable to other age groups, particularly older populations where a natural erosion of sleep quality and immunological function accompany the aging process. Thus, the narrower focus of the current investigation warrants expansion into broader subject populations with varied doses of CBD and more rigorous sleep analysis, such as polysomnography (PSG), to further confirm these findings. Finally, groups were not matched for biological sex, with the CB group containing two more females than the CN. Though research is limited, one study has demonstrated greater bioavailability in females compared with males when CBD is administered using MCT, as it was in the current investigation. Therefore, while we did implement methodology to optimize bioavailability in all of our participants through consuming CBD in a fed state, not having groups matched on biological sex should be noted as a limitation.

## 6. Conclusions

The daily consumption of 50 mg hemp-derived CBD resulted in improvements in the overall perceived quality of sleep as assessed using the LSEQ, with further improvements detected in the quality of sleep following eight weeks of CBD supplementation. Furthermore, significantly greater immune cell cytotoxicity directed at leukemia cells was observed between the CB and CN groups following the intervention, particularly at the 10:1 E:T cell ratio, as indicated by the reduced percentage of live K562 leukemia cells. These results collectively support the notion that low dose CBD supplementation may offer benefits in enhancing sleep quality in humans and improving immunosurveillance against cancer cells in situ.

## 7. Future Directions

The current investigation sets the stage for additional future investigations regarding CBD effects on sleep and immunological function. The improvements seen in perceived sleep quality following CBD administration are promising, and future work using PSG following CBD administration would be innovative in uncovering the effects of CBD on sleep architecture. Furthermore, increased CBD dosing, chronic CBD use, and additional CBD administration modes such as oral mucosal spray and topical gels in varying healthy and diseased populations will help strengthen the current body of clinical literature on CBD, and further explain its potential effects in the human population. Finally, with the differences in bioavailability between biological sexes, investigating the effects of CBD on males and females will help bolster the current body of literature and improve the accuracy of CBD dosing between sexes.

## Figures and Tables

**Table 1:** Participant Characteristics, Physical Activity Characteristics, Mental Health, Sleep, and Immunophenotype at Baseline.

**Variable**	**CB** **(Pre, *n =* 14)**	**CN** **(Pre, *n =* 14)**	**Male** **(*n =* 14)**	**Female** **(*n =* 14)**
Participant Characteristics				
Age (yr)	24.8 ± 5.5	27.1 ± 6.7	26.9 ± 5.1	25.0 ± 7.0
Sex (M/F)	6.0/8.0	8.0/6.0		
Height (cm)	161.7 ± 29.7	172.0 ± 9.5	176.5 ± 7.0	164.3 ± 8.0 **
BMI (kg/m^2^)	24.8 ± 3.6	24.7 ± 3.0	25.1 ± 2.7	24.4 ± 3.7
Weight (kg)	71.4 ± 15.6	72.8 ± 9.1	78.4 ± 11.0	65.7 ± 11.0 **
Lean Body Mass (kg)	55.9 ± 13.0	58.5 ± 9.7	66.9 ± 6.6	47.5 ± 4.7 **
Body Fat (%)	21.5 ± 8.1	19.3 ± 9.8	14.0 ± 5.8	26.8 ± 6.4 **
Physical Activity Characteristics				
Vigorous Activity (# Days)	4.8 ± 0.9	3.6 ± 1.8 *	4.3 ± 1.3	4.1 ± 1.8
Vigorous Activity (min)	61.4 ± 50.5	95.7 ± 116.2	77.9 ± 50.1	79.3 ± 119.0
Moderate Activity (# Days)	5.0 ± 2.1	3.9 ± 1.9	4.0 ± 2.2	4.9 ± 1.9
Moderate Activity (min)	46.1 ± 32.7	57.9 ± 35.3	53.9 ± 37.2	50.0 ± 31.6
Walking (# Days)	6.5 ± 0.9	6.2 ± 0.4	6.1 ± 1.6	6.6 ± 0.9
Time Spent Walking (min)	94.3 ± 97.4	93.9 ± 109.4	92.9 ± 111.2	95.4 ± 95.2
Sitting (hr)	5.6 ± 2.5	5.6 ± 2.1	6.1 ± 1.9	5.0 ± 2.5
Mental Health Measures				
**Variable**		**CB** **(Pre, *n =* 14)**	**CN** **(Pre, *n =* 14)**	
Beck’s Depression Inventory		5.3 ± 4.2	3.6 ± 3.1	
General Anxiety Disorder-7		7.9 ± 6.6	4.3 ± 4.7	
Piper Fatigue Scale		3.7 ± 1.6	2.6 ± 1.3	
Behavioral/Severity		2.8 ± 1.7	1.8 ± 1.2	
Affective Meaning		4.3 ± 1.7	3.2 ± 2.3	
Sensory		4.4 ± 1.7	3.1 ± 1.5	
Cognitive/Mood		3.7 ± 1.9	3.0 ± 1.8	
Quality of Life Index		19.0 ± 1.9	20.1 ± 1.5	
Health and Functioning		24.0 ± 3.8	25.3 ± 3.6	
Social and Economic		22.4 ± 4.1	24.4 ± 3.5	
Psychological/Spiritual		22.8 ± 4.6	25.5 ± 3.8	
Family		20.5 ± 7.7	24.8 ± 3.9	
Sleep Measures				
Minutes Asleep (min)		398.7 ± 49.5	377.0 ± 78.7	
Minutes Awake (min)		55.4 ± 10.5	49.3 ± 13.1	
Wake Episodes		28.4 ± 5.7	23.9 ± 8.9	
Time in Bed (min)		454.1 ± 57.5	426.4 ± 90.6	
Sleep Efficiency (%)		88.0 ± 1.5	88.9 ± 1.5	
LSEQ		41.8 ± 12.1	48.7 ± 15.7	
GTS		48.1 ± 14.0	53.3 ± 19.4	
QOS		31.3 ± 24.6	43.2 ± 18.2	
AFS		39.2 ± 19.7	42.8 ± 19.8	
BFW		44.2 ± 22.1	51.5 ± 19.4	
Immunophenotype				
CD3^−^CD56^+^ (NK Cells; %)		11.7 ± 6.6	10.8 ± 5.0	
CD3^−^CD56^++^ (CD56^bright^ NK Cells; %)		4.8 ± 3.0	2.8 ± 2.9	
CD3^+^CD56^+^ (NKT Cells; %)		5.8 ± 2.6	5.9 ± 4.2	
CD3^+^CD56^−^ (T-Cells; %)		51.7 ± 9.7	50.0 ± 9.2	

Values presented are mean ± SD. Abbreviations: CB—Cannabidiol, CN—Control, Pre—Pre-Intervention, yr—year, M/F—male/female, BMI—Body Mass Index, kg/m^2^—kg per meters squared, cm—centimeters, kg—kilograms, %—percent, # days—days performed activity in the past week, Activity min—minutes of activity per day, min—minutes, hr—hours per day, GTS—getting to sleep, QOS—quality of sleep, AFS—awake following sleep, BFW—behavior following wakening, NK—natural killer, NKT—natural killer T. *—indicates significant differences between groups (CB, CN; *p* < 0.05), **—indicates significant differences between groups (M, F; *p* < 0.05) at the pre-intervention time point.

**Table 2:** Participant Characteristics.

Variable	CB (Post, *n* = 14)	CN (Post, *n* = 14)	% Change Pre-Post	Mean% Change (CB)	Mean% Change (CN)
BMI (kg/m^2^)	24.9 ± 3.7	24.8 ± 3.0	0.5 ± 1.5	0.4	0.7
Weight (kg)	71.6 ± 15.9	73.3 ± 9.3	0.5 ± 1.5	0.3	0.7
Lean Body Mass (kg)	56.2 ± 13.3	58.4 ± 10.3	0.1 ± 2.2	0.5	−0.2
Body Fat (%)	21.4 ± 7.8	20.2 ± 10.2	3.6 ± 11.1	−0.1	4.3

Values presented are mean ± SD. Abbreviations: CB—Cannabidiol, CN—Control, Pre—Pre-Intervention, Post—Post-Intervention, % change—percent change of each participant pre to post, Mean %—change the average percent change for groups combined pre to post, BMI—Body Mass Index, kg/m^2^—kg per meters squared, kg—kilograms, *%*—percent.

**Table 3:** Mental Health Measures.

Variable	CB (Post, *n =* 14)	CN (Post, *n* = 14)	% Change Pre-Post	Mean% Change (CB)	Mean% Change (CN)
Beck’s Depression Inventory	5.6 ± 3.6	3.0 ± 3.5	30.2 ± 108.8	−1.3	−9.8
General Anxiety Disorder-7	5.8 ± 4.2	3.7 ± 4.3	195.7 ± 813.7	−32.3	−22.1
Piper Fatigue Scale	3.1 ± 1.7	2.3 ± 1.4	5.5 ± 62.8	−18.6	0.6
Behavioral/Severity	2.5 ± 2.0	2.1 ± 1.2	20.0 ± 77.8	−12.6	14.9
Affective Meaning	2.9 ± 2.0	2.7 ± 2.0	−1.8 ± 87.9	−33.3	−14.9
Sensory	3.7 ± 1.7	3.3 ± 1.8	7.9 ± 73.8	−17.7	4.8
Cognitive/Mood	3.6 ± 1.9	2.9 ± 1.7	23.7 ± 95.7	−1.9	−1.6
Quality of Life Index	19.4 ± 1.3	20.4 ± 1.4	2.0 ± 6.8	2.6	0.7
Health and Functioning	24.1 ± 2.5	26.5 ± 2.8	3.9 ± 12.5	0.6	4.7
Social and Economic	23.7 ± 5.0	23.4 ± 3.8	1.5 ± 15.4	5.8	−4.0
Psychological/Spiritual	24.8 ± 3.5	25.4 ± 3.8	7.2 ± 28.9	8.6	−0.5
Family	22.1 ± 5.9	26.3 ± 4.6	33.6 ± 147.5	7.8	6.3

Values presented are mean ± SD. Abbreviations: CB—Cannabidiol, CN—Control, Pre—Pre-Intervention, Post—Post-Intervention, % change—percent change of each participant pre to post, Mean % change—the average percent change for groups combined pre to post.

**Table 4:** Sleep Measures.

Variable	CB (Post, *n* = 14)	CN (Post, *n* = 14)	% Change Pre to Post	Mean% Change (CB)	Mean% Change (CN)
Objective Actigraphy Measures					
Minutes Asleep (min)	374.1 ± 55.4	395.3 ± 53.1	0.2 ± 17.8	−6.2	4.8
Minutes Awake (min)	53.8 ± 11.3	50.1 ± 8.1	1.5 ± 22.4	−2.7	1.6
Wake Episodes	24.4 ± 7.4	26.3 ± 6.9	−0.9 ± 30.4	−13.9	10.0
Time in Bed (min)	428.0 ± 64.4	445.3 ± 56.0	0.2 ± 17.5	−5.8	4.5
Sleep Efficiency (%)	87.6 ± 2.1	88.8 ± 1.7	−0.2 ± 2.2	−0.5	−0.1
Subjective Sleep Measures					
LSEQ	54.9 ± 7.3 **	55.6 ± 11.8	47.1 ± 121.4	27.8	14.3
GTS	53.5 ± 9.1	60.3 ± 15.7	54.9 ± 219.4	11.3	13.0
QOS	56.0 ± 21.5 **	53.8 ± 16.5	169.2 ± 310.9	79.2	24.5
AFS	60.4 ± 17.3 *	46.0 ± 21.4	76.0 ± 167.9	53.9	7.4
BFW	51.9 ± 17.8	58.6 ± 14.5	54.2 ± 129.0	17.3	13.7

Values presented are mean ± SD. Abbreviations: CB—Cannabidiol, CN—Control, Pre—Pre-Intervention, Post—Post-Intervention, % change—percent change of each participant pre to post, Mean % change—the average percent change for groups combined pre to post, LSEQ—Leeds sleep evaluation questionnaire, GTS—getting to sleep, QOS—quality of sleep, AFS—awake following sleep, BFW—behavior following wakening, min—minutes, mm—millimeters, min—minutes, %—percent. * Indicates significant difference (*p* < 0.05) pre to post intervention. ** Indicates significant difference (*p* < 0.01) pre to post intervention.

**Table 5:** Immunophenotype.

Variable	CB (Post, *n* = 14)	CN (Post, *n* = 14)	% Change Pre to Post	Mean% Change (CB)	Mean% Change (CN)
CD3^−^CD56^+^ (NK Cells; %)	11.6 ± 5.9	10.9 ± 4.4	8.8 ± 34.7	−0.4	1.5
CD3^−^CD56^++^ (CD56^bright^ NK Cells; %)	2.6 ± 2.9 *	0.7 ± 0.7 *	−47.1 ± 49.5	−46.5	−76.9
CD3^+^CD56^+^ (NKT Cells; %)	3.7 ± 1.9	4.9 ± 4.6	−25.2 ± 30.3	−36.7	−16.9
CD3^+^CD56^−^ (T-Cells; %)	54.1 ± 5.8	54.0 ± 10.0	8.0 ± 15.1	4.7	8.2

Values presented are mean ± SD. Abbreviations: CB—Cannabidiol, CN—Control, Pre—Pre-Intervention, Post—Post-Intervention, % change—percent change of each participant pre to post, Mean % change—the average percent change for groups combined pre to post, % percent, NK—natural killer, NKT—natural killer T. * Indicates significant difference (*p* < 0.05) pre to post intervention.

## Data Availability

The data that support the findings of this study are available from the corresponding authors upon reasonable request. There are no publicly archived datasets generated or analyzed during this study.
